# Anodal Transcranial Direct Current Stimulation Over S1 Differentially Modulates Proprioceptive Accuracy in Young and Old Adults

**DOI:** 10.3389/fnagi.2019.00264

**Published:** 2019-09-26

**Authors:** Toni Muffel, Franziska Kirsch, Pei-Cheng Shih, Benjamin Kalloch, Sara Schaumberg, Arno Villringer, Bernhard Sehm

**Affiliations:** ^1^Neuroplasticity and Motor Recovery Group, Department of Neurology, Max Planck Institute for Human Cognitive and Brain Sciences, Leipzig, Germany; ^2^Day Clinic for Cognitive Neurology, University Hospital, Leipzig University, Leipzig, Germany; ^3^Mind Brain Body Institute, Berlin School of Mind and Brain, Humboldt-Universität zu Berlin, Berlin, Germany; ^4^Center for Stroke Research Berlin (CSB), Charité – Universitätsmedizin Berlin, Berlin, Germany; ^5^International Max Planck Research School on the Life Course, Max Planck Institute for Human Development, Berlin, Germany; ^6^Institute of Psychology, Otto von Guericke University Magdeburg, Magdeburg, Germany; ^7^International Max Planck Research School on Neuroscience of Communication, Max Planck Institute for Human Cognitive and Brain Sciences, Leipzig, Germany; ^8^Faculty of Computer Science and Media, Leipzig University of Applied Sciences, Leipzig, Germany; ^9^Department of Neurology, Martin Luther University of Halle-Wittenberg, Halle, Germany

**Keywords:** tDCS, aging, proprioception, position sense, robotics, electrical field simulation

## Abstract

**Background:**

Proprioception is a prerequisite for successful motor control but declines throughout the lifespan. Brain stimulation techniques such as anodal transcranial direct current stimulation (a-tDCS) are capable of enhancing sensorimotor performance across different tasks and age groups. Despite such growing evidence for a restorative potential of tDCS, its impact on proprioceptive accuracy has not been studied in detail yet.

**Objective:**

This study investigated online effects of a-tDCS over S1 on proprioceptive accuracy in young (YA) and old healthy adults (OA).

**Methods:**

The effect of 15 min of a-tDCS vs. sham on proprioceptive accuracy was assessed in a cross-over, double blind experiment in both age groups. Performance changes were tested using an arm position matching task in a robotic environment. Electrical field (EF) strengths in the target area S1 and control areas were assessed based on individualized simulations.

**Results:**

a-tDCS elicited differential changes in proprioceptive accuracy and EF strengths in the two groups: while YA showed a slight improvement, OA exhibited a decrease in performance during a-tDCS. Stronger EF were induced in target S1 and control areas in the YA group. However, no relationship between EF strength and performance change was found.

**Conclusion:**

a-tDCS over S1 elicits opposing effects on proprioceptive accuracy as a function of age, a result that is important for future studies investigating the restorative potential of a-tDCS in healthy aging and in the rehabilitation of neurological diseases that occur at advanced age. Modeling approaches could help elucidate the relationship between tDCS protocols, brain structure and performance modulation.

## Introduction

Proprioception – the perception of body position and movement – is a vital function for daily activities including postural stability and reaching movements (Findlater et al., 2016), which declines in the course of aging (Stelmach and Sirica, 1986; Adamo et al., 2007; Herter et al., 2014; Hughes et al., 2015). Both changes in the peripheral and central nervous system have been associated with this decline, including decreases in muscle spindle sensitivity (Kim et al., 2007) and diameter (Kararizou et al., 2005) as well as atrophy in the postcentral gyrus, parietal and insular cortices (Good et al., 2001), which all are regions associated with proprioceptive processing (Goossens et al., 2019). Among cortical regions, the primary somatosensory cortex (S1) is the most important area to process proprioceptive information (Behrens et al., 2003; Findlater et al., 2016; Armenta Salas et al., 2018). On a functional level, Goble et al. (2012a) showed largely overlapping functional networks involved in proprioception in young (YA, <35 years) and older healthy adults (OA, >65 years), but reduced activity of the putamen in OA associated with poorer proprioceptive performance. Behaviorally, age-related changes in proprioception were assessed using an arm position matching (APM) task with occluded vision, showing that OA not only exhibited lower overall proprioceptive accuracy than YA, but also increased movement duration and frequency of speed peaks, both indicative of elevated uncertainty during task execution (Adamo et al., 2007; Herter et al., 2014). Hence, across the lifespan, proprioceptive deficits appear to accumulate gradually, with YA exhibiting highest accuracy, which decreases in middle aged adults (35–50 years), and is lowest in OA (Goble, 2010; Van de Winckel et al., 2017). Impaired proprioceptive processing is associated with an increased incidence of falls in OA (Lord et al., 1999) and inaccurate performance in reaching movements (Adamo et al., 2007). Therefore, proprioceptive deficits may result in detrimental consequences for daily activities, particularly in the late life phase. The clinical relevance of this problem is stressed by the fact that neurological diseases, such as stroke, often manifest at higher age, when a decline in proprioception additionally worsens symptoms and hampers sensorimotor recovery in the presence of such conditions. About 85% of acute stroke patients and approximately 50% of patients with stroke in the chronic phase suffer from motor deficits (Rathore et al., 2002; Carlsson et al., 2018), while up to 60% of patients also exhibit somatosensory and proprioceptive deficits (Connell et al., 2008; Carey and Matyas, 2011; Lima et al., 2015; Rand, 2018). Here, proprioceptive deficits are associated with motor deficits of the affected upper extremity (Rand, 2018).

Despite these clinical implications, it is yet unknown whether or how proprioception can be improved by means of interventional methods such as transcranial direct current stimulation (tDCS). On a physiological level it was shown that tDCS modulates the proprioceptive afferent system by changing the excitability of projections to propriospinal neurons (Bradnam et al., 2011; McCambridge et al., 2014). In this study, we targeted the primary somatosensory cortex (S1) as the primary cortical area to process proprioceptive information (Behrens et al., 2003; Findlater et al., 2016) in order to modulate behavior. While tDCS has been frequently applied over M1 to facilitate learning and cortical excitability (Buch et al., 2017), less is known about somatosensory tDCS and its potential to modulate the accuracy of sensory perception. Ragert et al. (2008) demonstrated an improvement of tactile spatial discrimination induced by anodal tDCS (a-tDCS) over S1 in YA. Similar findings were demonstrated with a different (bihemispheric) electrode montage over S1 (Fujimoto et al., 2014) and with vibrotactile discrimination (Labbe et al., 2016). Up to now, there is only one study investigating the effects of tDCS on somatosensation in OA (Zhou et al., 2018). In this study, vibratory thresholds at the foot sole were decreased during a-tDCS over S1 in OA, while no young control group was assessed. To our knowledge, there are no studies investigating the differential effects of tDCS on proprioception in YA and OA.

Various mechanisms contribute to changes in brain structure and function (Jagust, 2013) which could explain differential responses to tDCS in the two age groups. Apart from aging-related changes in excitation and inhibition rates (Cheng and Lin, 2013; Legon et al., 2016) or the reorganization of cortical representations/maps (Cabeza, 2002), changes in brain morphology (e.g., cortical thinning) might determine intensity and distribution of electrical fields (EF) and thereby the effect of tDCS (Mahdavi et al., 2018). Simulations of EF are gaining increasing importance when assessing individual or group-wise differences in response to tDCS. A recent study has established a direct connection between the tDCS-induced field strength in SM1 and modulations of brain connectivity and neurotransmitter concentrations in YA (Antonenko et al., 2019). However, individual simulations and their relationships to induced modulations on a behavioral level have not been compared between YA and OA so far.

In the present study, we investigated (1) possible effects of a-tDCS over S1 on proprioceptive accuracy in YA and OA and (2) the strength and distribution of the induced electrical fields (EF) in these groups based on modeling of individual brain structure. We hypothesized to elicit differential tDCS-induced behavioral changes as a function of age. Specifically, we expected that a-tDCS has only little to no measurable influence on performance in the YA group, since their proprioceptive accuracy may be physiologically optimized, resulting in a ceiling level performance. In OA, however, we expected lower baseline performance in proprioceptive accuracy and a more pronounced positive effect of a-tDCS, as compared to the YA group. Furthermore, we expected the individually modeled field strengths to be related to performance changes and to differ between groups, based on individual brain morphologies.

## Materials and Methods

### Participants

Participants were recruited from the in-house database of the Max Planck Institute for Human Cognitive and Brain Sciences in Leipzig. Health status was assessed by a medical examination prior to study enrolment. Persons were excluded from participation if they had contraindications for tDCS or any psychiatric or neurological disorders. In total, 45 participants were included and allocated to the experimental groups: “young adults” (YA, *n* = 21, 10 females) for ages 18–35 (mean age: 27.0 ± 2.4 years) and “old adults” (OA, *n* = 24, 12 females) for ages 60 to 80 years (mean age: 69.4 ± 4.9 years). Only right-handed healthy adults were included in this study, as assessed by the Edinburgh Handedness Inventory (Oldfield, 1971). Sex was distributed equally between groups (χ^2^(_1_, _45_) = 0.03, *p* = 0.87). The study was approved by the Ethics Committee of the University of Leipzig and written informed consent was obtained from all participants after extensive briefing prior to study enrolment and in accordance with the Declaration of Helsinki.

### Experimental Design and Procedure

**FIGURE 1:**
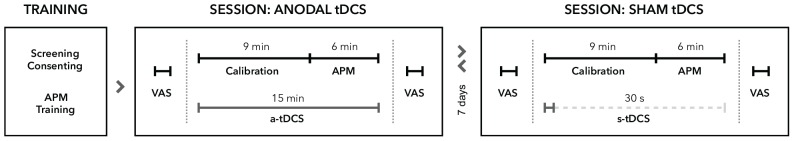
**Experimental procedure**. All participants underwent neurological examinations and task familiarization before study enrolment. Proprioceptive performance was assessed in two different sessions separated by at least 7 days. In each session, participants rated their levels of attention, wakefulness and pain on visual analog scales (VAS) before and after tDCS. All participants received either a-tDCS (15 min) or sham tDCS (30 s) concurrent to robotic measurements of proprioception by means of the arm position matching task (APM). In both conditions, stimulation commenced at the beginning of the calibration process for the robotic system. The order of stimulation was pseudo-randomized and balanced for all participants. Assessments of proprioceptive performance always started 9 min after stimulation onset.

Age-dependent effects of a-tDCS on proprioceptive accuracy were tested using a double-blind, cross-over design with the factors *age group* (YA vs. OA) and *stimulation condition* (sham vs. a-tDCS). For familiarization, all participants underwent a training session prior to testing (Figure 1). Stimulation effects were assessed on two individual days separated by one week (mean pause = 7.2 ± 0.4 days) to avoid carry-over effects. The experimental procedures were identical on both days except for the applied tDCS type (sham vs. a-tDCS), the order of which was counterbalanced across participants. The experiment was carried out by two experimenters: one performed all measurements, interacted with the participants and was blinded for the stimulation type, the other assisted during electrode placement and operated and supervised the DC stimulator.

Testing sessions began with a short questionnaire regarding tDCS safety and 10-point visual analog scales (VAS) for attention, wakefulness and pain. After tDCS preparations (described in the next section), the exoskeleton (described in section “Proprioceptive Assessment”) was mechanically adjusted to the participant and individual adjustment parameters were saved for the second test session. After tDCS was turned on, the robotic system was calibrated to account for individual limb-segment length and participants were again provided with the task instructions. The proprioceptive task started exactly 9 min after stimulation onset.

### Transcranial Direct Current Stimulation

**FIGURE 2:**
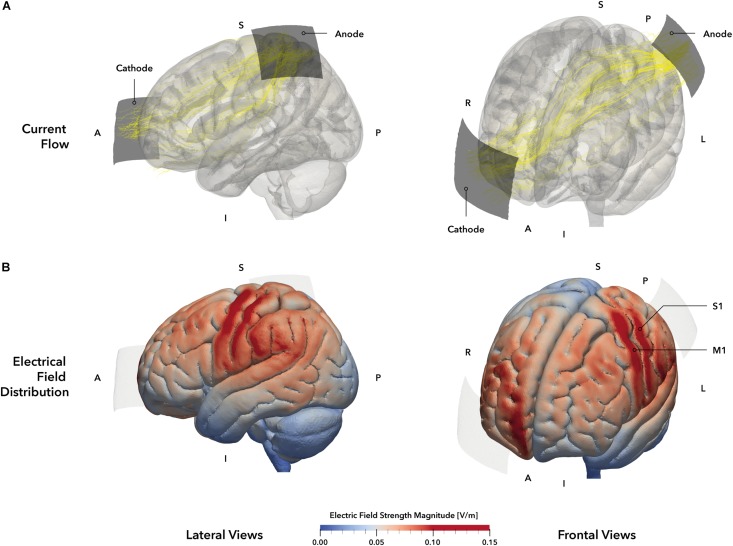
**tDCS montage and electric field simulation**. **(A)** Electrode montage and simulated current flow. Stimulation electrodes are displayed in dark gray. The anode was positioned over the primary somatosensory cortex (S1, 5 × 5 cm^2^) based on the 10–20 system and the cathode (5 × 7 cm^2^, blue) was placed over the contralateral orbit. Yellow lines represent the main current trajectories obtained from simulation. **(B)** Simulated electric field distribution. The tDCS setup of our study was simulated on the MNI152 head template, allowing a descriptive evaluation of electrical field distribution and strength. The simulation indicates electric field maxima in the left primary motor (M1) and somatosensory (S1) cortices (darker red areas), as well as weaker electrical fields in the left frontal premotor cortex and superior and inferior parietal lobules (brighter red areas). The right prefrontal areas beneath the cathode also exhibit a stronger electrical field strength. Regions within the gray matter volume with an electrical field strength of 0.1 V/m or higher were defined as stimulation hot spots and highlighted in red. Electrodes are displayed in pale gray. *A* = anterior, *I* = inferior, *L* = Left, *P* = posterior, *R* = right, *S* = superior.

The 10–20 system (Oostenveld and Praamstra, 2001) was used for electrode positioning. The anode (5 × 5 cm^2^) was placed over S1 (C3’, approximately at 10% of the nasion-inion distance posterior C3, according to Ragert et al., 2008; see Figure 2). The cathode (7 × 5 cm^2^) was placed over the contralateral orbit (Nitsche et al., 2008; Woods et al., 2016). After scalp preparation, electrodes were positioned using Ten20 conductive electrode paste (Weaver and Company, Aurora, CO, United States) and supported by a rubber band for good adhesion and low impedances, which were consistently held below 10 kΩ.

Stimulation setup and parameters were based on previously published protocols that reported facilitatory effects of a-tDCS on somatosensory function (Ragert et al., 2008) and were adjusted for the requirements of this experiment. In the *verum* condition (a-tDCS), 1 mA of direct current was delivered for 15 min. In the *placebo* condition (sham), 1 mA was applied for 30 s (Gandiga et al., 2006). In both conditions, current was faded in and out for 30 s each. Current was delivered by a battery-driven DC stimulator (Neuroconn GmbH, Ilmenau, Germany).

To verify the chosen electrode setup for the stimulation of S1, we performed a simulation of the current distribution and current flow for the mentioned tDCS setup (Figure 2A) using SimNIBS 2.1 (Thielscher et al., 2015) and the included MNI head model. According to the simulation, the selected setup elicited a strong EF within the postcentral (S1) and precentral (M1) gyri and the inferior parietal lobule (Figure 2B), representing the area underneath the anode. Moreover, a second smaller field maximum could be identified at the contralateral prefrontal interhemispheric ridge, located underneath the cathode. Accordingly, the applied setup should have resulted in a unilateral stimulation of the primary sensorimotor cortices with a larger involvement of parietal brain regions.

### Proprioceptive Assessment

**FIGURE 3:**
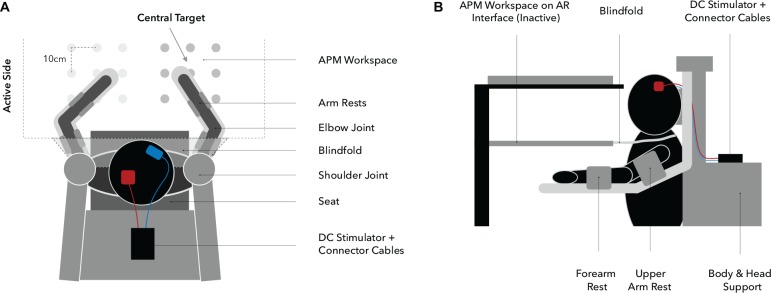
**Experimental setup** (**A:** top view, **B:** side view). Participants are seated in the exoskeleton with their arms supported against gravity by two arm rests, allowing only planar movements. Targets are spaced 10 cm apart from each other and the central target, determining the overall positioning of the workspace, which is adjusted for each participant individually at 90° elbow flexion and 30° horizontal shoulder abduction **(A)**. Visual information regarding upper extremity position are withheld through a closable mirror and an apron which is wrapped around the shoulders **(B)**.

**FIGURE 4:**
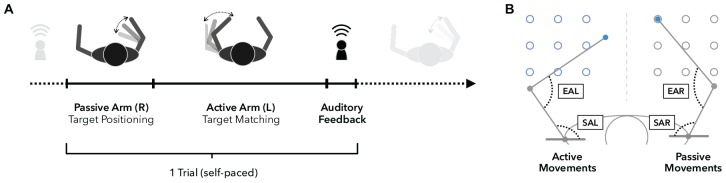
**Arm position matching task**. **(A)** Timeline of a single trial. Upon trial start, the right arm is moved passively by the robot to one of nine possible targets (see also panel **B**), while vision is blocked. Participants are instructed to focus on the perception of the right arm position in order to move their left arm to mirror-match the position of the right arm as accurately as possible. Participants notify the experimenter when they subjectively reached the accurate position to end one trial and start the next one from that end position. **(B)** Workspace and accuracy measures. The workspace contains nine targets for the left (*active side*) and right (*passive side*) arm. Absolute errors (AE) are defined as the absolute spatial distance in (cm) between left and right hand positions. Elbow angle errors (EAE) represent the absolute angular differences in flexion angles between the right elbow angle (EAR, passive) and left elbow angle (EAL, active). Similarly, shoulder angle errors (SAE) are defined as the absolute angular differences in horizontal shoulder abduction between the right (SAR, passive) and left (SAL, active) shoulder joints.

Behavioral assessments were performed using the KINARM exoskeleton (BKIN Technologies Ltd., Kingston, ON, Canada; Figure 3), a robotic system recording elbow and shoulder movements at a spatial resolution in the millimeter range and at temporal resolution of 1000 Hz (Ball et al., 2009). To investigate proprioceptive accuracy, the APM task (Dukelow et al., 2010, 2012; Herter et al., 2014; Kenzie et al., 2014; Semrau et al., 2015a, b), measuring static joint position sense, was used. During a single trial (Figure 4A), the robot moves the participant’s right arm to one of nine possible positions (denoted *targets*, Figures 3, 4B) in the workspace. This movement follows a straight path and is passive for the participant (therefore the *passive side*). Participants are instructed to actively mirror-match the end position of the right arm displacement with their left arm, while visual perception of hand, elbow and shoulder is blocked. Accordingly, the left side is denoted *active side*. If participants consider their performance accurate, they notify the experimenter and the trial ends (Figure 4A). Trials are self-paced and participants are instructed to focus on accuracy only. A new trial starts with a passive movement from the current arm position to a different target. The complete APM assessment consists of 54 trials, with each of the nine targets (Figure 3) being approached six times in a random order.

### Data Analysis

If not otherwise stated, all analyses were performed in SPSS 18 (SPSS Inc., Chicago, IL, United States). An alpha value of 5% was used for all inferential statistics.

#### Visual Analog Scales

10-point visual analog scales (VAS) for attention (1 not at all attentive – 10 very attentive), wakefulness (1 very tired – 10 very awake), and pain (1 no pain – 10 strong pain) were obtained before (pre) and after (post) stimulation. Pre-post changes for each VAS were calculated and then compared between stimulation conditions using paired-sample *t*-tests. All participants tolerated the stimulation well and there were no adverse events during experimental procedures. During stimulation, no significant pre-post changes occurred neither in attention (*t*(_44_) = –0.57, *p* = 0.57), nor in wakefulness (*t*(_44_) = 0.38, *p* = 0.71), nor in pain (*t*(_44_) = 1.43, *p* = 0.16). There were no significant differences (pre vs. post stimulation) between conditions in levels of attention (pre: *t*(_44_) = 0.00, *p* = 1.00; post: *t*(_44_) = 0.31, *p* = 0.76), wakefulness (pre: *t*(_44_) = 0.70, *p* = 0.95; post: *t*(_44_) = –0.86, *p* = 0.39), or pain (pre: *t*(_44_) = 1.35, *p* = 0.18; post: *t*(_44_) = 1.07, *p* = 0.29). There were no significant differences in pre or post VAS between age groups.

#### Blinding

Both participants and the primary experimenter were blinded regarding the applied stimulation condition and were only debriefed after the second testing session. To assess the efficiency of blinding, participants had to indicate whether or not they thought they had been stimulated after each session and to provide a measure of their certainty. Blinding efficiency was then assessed using the McNemar test, which revealed no significant differences in the reported perception of stimulation between a-tDCS and sham (*x*^2^ = 3.32, *p* = 0.19). Out of 45 participants, 48.9% perceived stimulation correctly in the a-tDCS and 42.2% in the sham condition. The mean reported certainty of the participants was 6.4 ± 2.6 (scale: 1 unsure – 10 absolutely certain) during a-tDCS and 6.1 ± 2.6 during sham. There was no difference between conditions regarding the certainty (*t*(_44_) = –0.82, *p* = 0.42). We conclude that the blinding approach was effective for two reasons: firstly, only about half of the participants correctly identified the applied protocol and secondly, the mean certainty was moderate – participants were either absolute sure or tended to select a rating around 5 to indicate that they are guessing.

#### Learning Effects

To assess potential learning from the first to the second session, irrespective of the applied stimulation, we compared the performance between the two sessions. None of the outcome measures (AE, Var, EAE, SAE) differed significantly between the first and second session. We therefore conclude that the behavioral results are specific to the applied a-tDCS.

#### Task Duration

The APM task was designed to measure static limb position sense and participants are instructed to focus on position matching accuracy only, effectively making trials self-paced. Although this approach should remove temporal aspects like a speed-accuracy-tradeoff, we also considered the total duration of task execution here. APM task durations did not differ between the two *stimulation conditions* (main effect: *F*(_1_, _43_) = 3.50, *p* = 0.557) nor *groups* (main effect: *F*(_1_, _43_) = 0.118, *p* = 0.73).

#### Proprioceptive Performance (Accuracy and Variability)

The performance in position matching was quantified by calculating the mean absolute difference between passive and active elbow and shoulder joint angles (elbow angle error [EAE] and shoulder angle error [SAE], respectively; Figure 4B) at the end of each trial (Adamo et al., 2007; Fuentes and Bastian, 2010). Both parameters are a measure of the participant’s perceived arm position. In addition, the standard deviation of both accuracy parameters across all trials (V_EAE_ and V_SAE_, respectively) was calculated to assess the variability of performance. All four parameters were calculated for the a-tDCS and sham conditions using MATLAB R2017b (MathWorks, Inc., United States). Repeated measures analyses of variances (RM-ANOVAs) with *stimulation condition* (a-tDCS vs. sham) as within-participant factor and *age group* (YA vs. OA) as between-group factor was used. *Post hoc* paired *t*-tests were applied when necessary to compare means between samples, *p*-values were corrected for multiple comparisons using the Bonferroni method.

#### Individual EF Simulations

To assess the distribution and magnitude of the induced EF between participants, we conducted individual EF simulations using SimNIBS 2.0 (Thielscher et al., 2015). For 35 participants (16 YA, mean age: 27.12 ± 2.5 years; 19 OA, mean age: 70.2 ± 4.2 years), high-resolution T1-weighted anatomical brain scans were procured from the participants database of the Max Planck Institute for Human Cognitive and Brain Sciences in Leipzig. Scans were chosen to be in temporal proximity to the proprioceptive assessments (mean difference: 4.6 ± 8.3 months).

Individual volumetric head models were generated using the *mri2mesh* script included in SimNIBS. The final head models exhibited five compartments representing the scalp, skull, cerebrospinal-fluid (CSF), gray matter and white matter. Due to an inconsistent estimation of the skull thickness, we pursued an alternative approach uncoupled from *mri2mesh* for the segmentation of the scalp, skull and CSF for all subjects. A multi-atlas-based approach was employed which computed a head segmentation in a majority voting process (Kalloch et al., 2019), resulting in a more robust representation of the thickness of the skull and CSF. Aside from this change in the segmentation routines, any further processing was done utilizing the SimNIBS *mri2mesh* pipeline again.

After obtaining the head model, we geometrically defined and positioned the electrodes in the SimNIBS graphical user interface. The electrodes were modeled as 5 × 5 cm^2^ (anode) and 7 × 5 cm^2^ (cathode) patch electrodes with an additional gel layer and a power connector. The positions of the electrodes were determined using our own customized plugin for the 3D modeling software Blender (Blender Foundation, Blender Institute, Amsterdam^1^). By interactively marking fiducial points (nasion, inion and both tragi) on the scalp surface of the individual subject, a 10–20 coordinate grid was generated which allowed a reliable electrode positioning. The anode was positioned at 10% of the nasion-inion distance behind C3 [as described in Ragert et al. (2008)], the cathode at Fp2. Determined electrode coordinates were then used for electrode placement in SimNIBS. All current outlets were modeled at the posterior sides of the electrodes. For all electrode placements, head measurements obtained during the actual proprioceptive assessments were used to aid and validate the virtual placement. We used the standard isotropic conductivities for the compartments as predefined in SimNIBS:

$$\sigma_{s\,k\,i\,n}=0.465\,\frac{s}{m},\sigma_{s\,k\,u\,l\,l}=0.01\,\frac{s}{m},$$

$$\;\,\sigma_{C\,S\,F}=1.654\,\frac{s}{m},\sigma_{G\,M}=0.275\,\frac{s}{m},$$

$$\;\,\sigma_{W\,M}=0.126\,\frac{s}{m},\sigma_{e\,l\,e\,c\,t\,r\,o\,d\,e}=1.0\,\frac{s}{m}.$$

Finally, the electrical field strength was calculated for the entire head volume.

For subsequent analyses and visualization, data were processed in ParaView 5.4 (Sandia National Laboratory, Kitware Inc., and Los Alamos National Laboratory). EF strength values were extracted from 3 predefined regions of interest (ROI) within S1, our main target area, as well as M1 and the contralateral frontal lobe, to serve as control areas on an individual basis. We defined the S1 and M1 masks anatomically informed and based on the Human Brainnetome Atlas (Fan et al., 2016) in MNI space. The S1 mask was created by the intersection of the postcentral gyrus mask of the atlas and a 3 cm diametric sphere around the upper limb coordinate in S1 (MNI coordinates: –40, –27, 54; Mayka et al., 2006). The M1 mask represents the intersection of the precentral gyrus mask of the atlas and a sphere of, again, 3 cm at the upper limb coordinate in M1 (MNI coordinates: –37, –25, 64; Mayka et al., 2006). To transform these two anatomically defined ROI masks from MNI space into the individual space of the subject MRI data, we spatially normalized the skull-stripped brain MRI data first by a linear registration using FSL FLIRT (Jenkinson and Smith, 2001; Jenkinson et al., 2002), followed by a non-linear registration in ANTs (Avants et al., 2011) to the MNI152 1 mm brain template. We then co-registered the ROI mask into the individual space by inverting the before determined normalization transformations. The third ROI was defined relative to the individual position of the frontal electrode of each subject. We projected the coordinate of the center of mass of the frontal electrode to the cortical gray matter compartment and used the resulting coordinate as the center of the 3 cm spherical frontal ROI. Since these operations were done in subject space, no transformation was necessary in this case. From each of the ROI masks in subject space, we extracted only the portion of the electrical field data of the head model that was located within both the respective ROI mask and the gray matter volume compartment per subject (Figure 6B) and computed its mean value. Additionally, we co-registered the field data of the entire head model of each subject into MNI space again using FLIRT and ANTs and the before determined normalization transformations to be able to visualize the EF pattern at the cortical surface on a group-level, as illustrated in Figure 6A.

The extracted values were compared between groups using an independent samples *t*-test. To assess a potential relationship between the induced EF and behavioral changes through tDCS, a change score was calculated for both mean outcome parameter using the formula %Δ_EAE_ _or SAE_ = 100 ^∗^ (EAE or SAE _*anodal*_ – EAE or SAE _*sham*_)/EAE or SAE _*sham*_ using either EAE or SAE values for computations, respectively (Ameli et al., 2009). Higher values indicate inferior performance due to an increase in error rate through a-tDCS. The change scores were then correlated to the field strength values in each group using Spearman’s linear correlation.

## Results

### Proprioceptive Performance (Accuracy and Variability)

#### Main Effect *Stimulation Condition*

Performance in matching accuracy (EAE: *F*(_1_, _43_) = 0.476, *p* = 0.49, η^2^ = 0.01; SAE: *F*(_1_, _43_) = 2.711, *p* = 0.11, η^2^ = 0.06) or variability (V_EAE_: *F*(_1_, _43_) = 0.014, *p* = 0.91, η^2^ < 0.001; V_SAE_: *F*(_1_, _43_) = 0.229, *p* = 0.64, η^2^ = 0.005) did not differ significantly between the two *stimulation conditions* across both groups.

#### Main Effect *Age Group*

The two groups did not differ significantly in matching accuracy (EAE: *F*(_1_, _43_) = 2.89, *p* = 0.10, η^2^ = 0.06; SAE: *F*(_1_, _43_) = 2.61, *p* = 0.11, η^2^ = 0.06) or variability (V_EAE_: *F*(_1_, _43_) = 1.31, *p* = 0.26, η^2^ = 0.03; V_SAE_: *F*(_1_, _43_) = 2.84, *p* = 0.10, η^2^ = 0.06).

#### Interaction Effect

**FIGURE 5:**
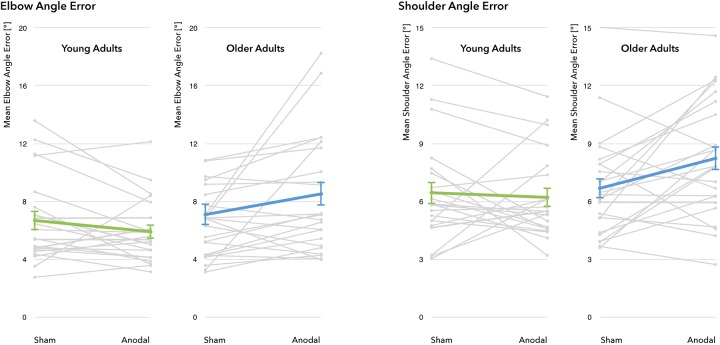
**Performance changes through a-tDCS**. No significant main effect for the factors *stimulation condition* (sham vs. a-tDCS) and *age group* (young adults vs. older adults) was identified. For all four outcome measures, there was a significant interaction between *age group* and *stimulation condition*, indicating differential effects of a-tDCS in young and old adults. Gray lines represent individual change trajectories for EAE and SAE in each group, thicker lines represent group means (error bars provided as ±1 standard error of the mean).

A differential effect of a-tDCS on performance was demonstrated by a significant interaction between *age group* and *stimulation condition* for all four outcome parameters (EAE: *F*(_1_, _43_) = 5.22, *p* = 0.027, η^2^ = 0.11; SAE: *F*(_1_, _43_) = 5.07, *p* = 0.029, η^2^ = 0.11; V_EAE_: *F*(_1_, _43_) = 6.77, *p* = 0.013, η^2^ = 0.14; V_SAE_: *F*(_1_, _43_) = 6.79, *p* = 0.013, η^2^ = 0.14), with YA exhibiting slight reductions in EAE and SAE whereas OA showed marked increases through stimulation (see Figure 5 for individual modulations and group means). *Post hoc t*-tests revealed that during sham, both age groups performed on a similar level (mean EAE*_*YA*_* = 6.68 ± 3.04°, mean EAE*_*OA*_* = 7.09 ± 3.68°, *t*(_43_) = 0.41, *p* = 0.87; mean SAE*_*YA*_* = 6.45 ± 2.67°, mean SAE*_*OA*_* = 6.68 ± 2.64°, *t*(_43_) = 0.289, *p* = 0.774). During stimulation, age groups diverged significantly in performance (EAE: *t*(_36__.__8_) = 2.687, *p* = 0.011; SAE: *t*(_43_) = 2.498, *p* = 0.016): while YA slightly improved under a-tDCS (mean EAE_YA_ = 5.91 ± 2.27°, mean SAE_YA_ = 6.21 ± 2.24°), the performance of the OA decreased (mean EAE_OA_ = 8.52 ± 4.09°, mean SAE_OA_ = 8.23 ± 3.05°).

### Individual EF Simulations

**FIGURE 6:**
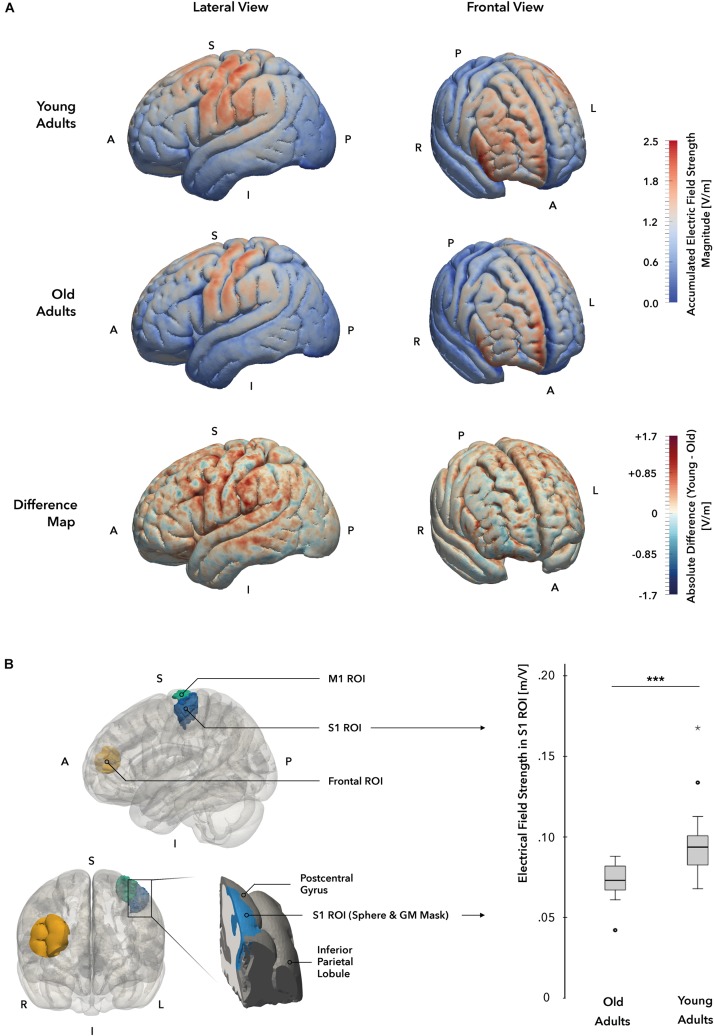
**Individual electrical field simulations**. **(A)** Whole-brain electrical field distribution. While the general pattern of electrical field distributions is similar in both groups, the difference map suggests that electrical fields at the cortical surface are stronger in young (YA; displayed in red) as compared to the old adults (OA; displayed in blue) group. **(B)** Electrical field strength in target region S1. A cortical gray matter ROI mask (marked in blue, left panel) of the hand and elbow region of S1 was used to compare field strength magnitudes between groups. A significant difference in electrical field strengths could be identified, with YA receiving higher field values in the ROI mask. *A* = anterior, *I* = inferior, *L* = Left, *P* = posterior, *R* = right, *S* = superior.

The simulation of individual EF indicated significantly stronger EF strengths in the primary target region S1 in the YA group when compared to the OA group (*t*(_33_) = –3.80, *p* = 0.001, two-sample *t*-test, Figure 6B). The general pattern of EF distributions across participants is similar when compared between groups, however, the YA group showed markedly higher field strengths and a wider distribution of elevated EF values in both primary sensorimotor (S1 ROI mean: YA = 0.097 ± 0.025 V/m, OA = 0.074 ± 0.08 V/m, M1 ROI mean: YA = 0.097 ± 0.024 V/m, OA = 0.074 ± 0.008 V/m) and frontal regions (Frontal ROI mean: YA = 0.096 ± 0.023 V/m, OA = 0.077 ± 0.012 V/m) of the cortex (Figure 6A). In comparison, the OA group also showed field strength maxima in S1 and M1, albeit less intense, but only low EF strengths were detected in supplementary motor area, premotor cortex and parietal areas. Likewise, the frontal field appeared to focus along the frontopolar interhemispheric ridge and shared the general pattern as in the YA group, but showed otherwise less intense field strengths within the distribution area. In both age groups, no significant correlation between the induced EF strength and the behavioral change scores could be established at a corrected *p*-level < 0.05.

## Discussion

We investigated the effect of a-tDCS over S1 on proprioceptive performance in YA and OA. We showed that tDCS differentially modulates task performance in an age-dependent manner: while at baseline there was no statistical difference between the two age groups regarding their proprioceptive matching accuracy, a-tDCS induced a slight performance increase in the YA group and a more prominent performance decrease in the OA group, leading to a significant difference in performance under stimulation. Additionally, the same stimulation setup induced different EF in the two groups, with YA receiving significantly higher EF strengths in S1 and in control areas. However, a direct linear relationship between induced field strength and the behavioral modulation could not be identified in our sample. Our findings highlight the impact of aging on the modulatory influence of tDCS on proprioceptive performance. Future studies are needed to elucidate age-related neuroanatomical, neurophysiological and cognitive-behavioral factors that might underlie these differential results.

Even during healthy aging individuals often undergo a decline in perceptual, motor and cognitive functions. A better knowledge of underlying mechanisms and new ways to prevent or even restore this functional decline is a major goal of current aging research. We here focused on proprioceptive function, since this modality is a prerequisite for successful motor control. Age-related decline of proprioception has been shown to impair locomotor function (Berard et al., 2012), balance (Maki and McIlroy, 2006; Kaminski et al., 2013) and goal-directed movements of the upper limbs (Sarlegna, 2006). In addition, proprioceptive deficits significantly hamper the recovery process of deficient motor function in the presence of neurological diseases.

Despite the clinical significance, it remains a challenge to assess proprioceptive performance in a sensitive and specific way. Available tests include ipsilateral and contralateral matching, that require active motion, as well as psychophysical threshold methods of purely passive movements. These methods represent measures, that, despite, their target modality proprioception, also inherently cover other aspects of neural processing. For example, ipsilateral and contralateral matching methods both require active motion and consequently are influenced by additional sensorimotor processes (Elangovan et al., 2014). Furthermore, ipsilateral matching and psychophysical threshold hunting require a higher demand on working memory, which has been found to influence the results especially in elderly populations (Goble et al., 2012b). Since the target of this study was to investigate age-related processes, we used a robotic APM task which assesses proprioceptive performance by matching interlimb accuracy with low working memory demand. This task has been used extensively in previous studies and has been shown to be sensitive to proprioceptive deficits in both aging and clinical populations (Dukelow et al., 2010; Herter et al., 2014). In the present sample, however, a significant difference in matching performance could not be established, in contrast to both the hypothesis of this study and previous findings (Stelmach and Sirica, 1986; Adamo et al., 2007; Herter et al., 2014). The reason to this could be that the sample size used in the current study was not large enough to establish age-related performance differences (in the absence of tDCS). Furthermore, differences regarding the influence of aging on proprioceptive accuracy across studies are likely reflecting the diversity of experimental designs. For instance, previous studies have assessed proprioception with other tasks and outcome measures that are likely more rater-dependent and cognitively demanding, as discussed above, than the robotic approach presented here (e.g., joint angle error vs. location of the hand; Herter et al., 2014), which could have resulted in more apparent group differences. Furthermore, such differences are also more prominent and thus easier detectable in the lower limb (Proske and Gandevia, 2012; Henry and Baudry, 2019), but the upper limb was assessed in the present study. For instance, age-related difference in the elbow matching study by Adamo et al. (2007) were on average below 2°.

In contrast to previous publications and our hypothesis (Ragert et al., 2008; Labbe et al., 2016; Summers et al., 2016), we did not show an overall beneficial effect of a-tDCS. Notably, these other studies used different experimental designs, including other assessments of somatosensory perception, and slightly different tDCS protocols, impeding a direct comparison between results. However, our findings do provide evidence that individual factors such as age might crucially influence both strength and directionality of tDCS effects (Heise et al., 2014; Craig and Doumas, 2017). In OA, findings of tDCS-induced modulations are not consistent across studies: while some report beneficial effects on behavioral performance (Learmonth et al., 2015; von Rein et al., 2015), others show negative responses to a-tDCS in learning tasks, such as dynamic balance learning (Kaminski et al., 2017). Our findings extend the notion of missing efficacy in OA and suggest that a-tDCS can actually impair function. Taken together, the healthy aging brain might respond fundamentally different to a-tDCS on a behavioral level.

While the underlying mechanisms are not yet understood, our result is interesting in the context of a recent study that investigated effects of tDCS on short intracortical inhibition, a measure that is considered to reflect GABAergic inhibition (Heise et al., 2014). While a-tDCS induced a release of inhibition in YA, an increase in inhibition was observed in OA. While such opposing effects were not demonstrated on a behavioral level in the same study, this result raises the notion that a-tDCS effects on the balance of excitation/inhibition in the cortex might be essentially different in YA vs. OA. Indeed, the “baseline” balance of excitation and inhibition is different between the two groups (Cabeza, 2002; Luebke et al., 2004) and could be one explanation of differential tDCS-induced effects between age cohorts. Another study used TMS to investigate temporal dynamics of responses after a-tDCS and showed that there is a delayed response on cortical excitability in OA (Fujiyama et al., 2014). Hence, both timing and direction of tDCS-induced neuroplastic changes vary with respect to age, a notion that might have contributed to the effects observed in our study.

Despite such local electrophysiological differences of tDCS effects, a complementary explanation could be that different brain networks are engaged in YA vs. OA while performing the same task (Cabeza, 2002; Zimerman et al., 2014). Therefore, the response to targeted stimulation of specific cortical areas might essentially vary as a function of age. For example, it is a well-described phenomenon that OA activate larger brain networks and recruit additional contra- and ipsilateral brain areas in order to perform a motor task similarly to YA (Ward, 2003; Seidler et al., 2010) and that such reorganizations are highly individual (Park and Reuter-Lorenz, 2009; Reuter-Lorenz and Park, 2014). While such evidence in the somatosensory domain is scarcer, one study showed activity increases in contralateral S1 and decreases in S2 and the cingular cortex during tactile stimulation in OA which correlated negatively with perceptual performance (Lenz et al., 2012; Brodoehl et al., 2013). In the context of our results, it is tempting to speculate that a stimulation of overactivated hubs in elderly might have interfered with the respective network and in consequence decreased performance.

A general limitation inherent to tDCS is the low spatial resolution. Hence, other areas than the primary target area might be responsible for the induced effects (Prehn and Floel, 2015). An alternative explanation of our findings might relate to the electrode setup. While the anode was attached over the left S1, the “reference” cathodal electrode was placed over the supraorbital region of the forehead, which represents the current standard for a-tDCS setups (Nitsche et al., 2008; Buttkus et al., 2011). It cannot be ruled out that also the cathode influenced cortical activity in the frontopolar cortex. To prevent this problem, we used an enlarged reference electrode to decrease current density and thereby render stimulation inefficient (Nitsche et al., 2008). However, the EF simulation showed high field strengths in the frontal pole and interhemispheric ridge. In the frontal ROI, which was selected as the focus point of the cathode here, no group-specific difference could be identified that could explain the detrimental behavioral effect in the OA group. In the present study, we chose a unilateral setup based on previously published protocols for S1 and M1 stimulation, as we sought to stimulate left S1 and investigate the modulatory effects of a-tDCS on proprioception in this area. However, our simulation showed a non-focal distribution of the induced EF in the whole sensorimotor system (see also next paragraph). The application of a more focused stimulation protocol, i.e., a high-definition or multi-electrode approach with a small anode and a Laplacian array of small return electrodes (e.g., Villamar et al., 2013), could have delivered stronger current loads to S1, thereby exerting stronger or different modulatory effects on proprioceptive accuracy, as highlighted in previous research investigating the effects of high-definition tDCS over M1 (Kuo et al., 2013). A possible mechanism of action that drives differential effects between the two approaches might be a selective interaction with center-surround inhibition in the sensorimotor system (Beck and Hallett, 2011).

With respect to the question of stimulation focality, we employed modeling of our electrode setup using high-resolution MRI-based head models to obtain EF maps. With this simulation approach, we showed that S1 was among the regions with the highest electric field strengths, supporting that with our stimulation setup the target brain structure S1 was reached. Furthermore, we showed differences in EF strengths between age cohorts: YA experience stronger EF strengths than OA both in S1 and in the other ROIs (M1 and frontal cortex). Since this was found in all areas tested, it might represent a general difference between YA and OA that needs to be considered when tDCS is applied to elderly cohorts. In line with recent findings (Im et al., 2018; Mahdavi et al., 2018; Thomas et al., 2018), this difference could be caused by age-related anatomical changes such as cortical thinning or atrophy, both inducing higher intracranial CSF volumes and, as a consequence, stronger current shunting. Moreover, skull thickness changes during healthy aging (Lillie et al., 2016) could further affect the induced EF. Indeed, it is plausible that the differential effect observed in the two age groups is reflective of a current-dosage-dependency, based on aforementioned age-related structural changes of the brain. If OA indeed receive weaker EF strengths overall, increasing the induced EF either by applying stronger current (e.g., 2 mA) or by increasing the current density under the anode (see also next paragraph) could potentially reverse the observed effect pattern. To test this in detail, a dose titration protocol could be applied, as commonly used in other areas of medicine and pharmacotherapy (e.g., Turnheim, 2003). However, the present study was designed to assess potential differential effects of a defined dose of a-tDCS in two age groups. It was not designed to investigate the differential effects of incremental a-tDCS dosages. The fact that no direct relationship between field strength and behavioral modulation was found might be explained by (i) latent age-related parameters other than the induced EF that mediate the relation between the variables tested in our study and (ii) the possibility, that other brain areas then the targeted S1 significantly contributed to the effects.

Future studies should also investigate the efficacy of other tDCS setups to modulate proprioception across the life span, as they might differentially interact with neurophysiological properties. For instance, although generally believed to have a dampening effect on cortical excitability (Nitsche and Paulus, 2000), cathodal tDCS has been shown to enhance motion perception in a visual tracking task (Antal et al., 2004), possibly interacting with a reduced signal-to-noise ratio in neural processing in the OA group (Hämmerer et al., 2013). Future studies should also investigate the effect of a bilateral S1 electrode setup (Fujimoto et al., 2014) specifically in OA, since it might interfere stronger with age-related interhemispheric activation changes (Mooney et al., 2018; Strauss et al., 2019). It is also possible that afferent sensory functions are modulated in a different way by means of classical tDCS protocols as compared to efferent motor functions. Especially when considering the folded architecture of the human cortex, hand and elbow areas in M1 and S1 are located at different positions on the surface of the precentral gyrus and the anterior ventral regions of the postcentral gyrus, respectively (Mayka et al., 2006), and are therefore differently oriented in relation to EF applied through a-tDCS (Fox et al., 2004). The peculiar relationships between cortical column orientation, induced EF and behavioral modulations need to be explored further.

Taken together, the results of the present study demonstrate for the first time that aging is a critical modulator of tDCS-induced changes in proprioceptive accuracy. The results of this study are important for future studies designed to develop tDCS as a therapeutic tool to enhance sensorimotor functions in OA or in patients with neurological diseases.

## Data Availability Statement

The datasets used and analyzed during the current study are available from the corresponding author upon reasonable request.

## Ethics Statement

The studies involving human participants were reviewed and approved by Ethics Committee of the University of Leipzig. The participants provided their written informed consent to participate in this study.

## Author Contributions

TM: conception and design of the work, data collection and procurement, data analysis and interpretation (all data), drafting the article, critical revision of the article. FK: conception and design of the work, data collection, data analysis and interpretation (behavioral data), drafting the article, critical revision of the article. P-CS: data collection, data analysis and interpretation (behavioral data), critical revision of the article. BK: data analysis and interpretation (simulations), drafting the article, critical revision of the article. SS: data analysis and interpretation (simulations), critical revision of the article. AV: conception and design of the work, supervision, drafting the article, critical revision of the article. BS: conception and design of the work, supervision, data interpretation (all data), drafting the article, critical revision of the article. All authors read and approved the final manuscript. Moreover, all authors agreed to be accountable for all aspects of the work.

## Conflict of Interest

The authors declare that the research was conducted in the absence of any commercial or financial relationships that could be construed as a potential conflict of interest.

## Footnotes

1 https://www.blender.org

## Abbreviations

ANOVA: analysis of variances
APM: arm position matching task
a-tDCS: anodal transcranial direct current stimulation
EAE: elbow angle error
EF: electrical field
M1: primary motor cortex
MNI: Montreal Neurological Institute
OA: old adult group
ROI: region of interest
S1: primary somatosensory cortex
SAE: shoulder angle error
SD: standard deviation
tDCS: transcranial direct current stimulation
VAS: visual analog scale
V_EAE_: variability (standard deviation) of the elbow angle error
V_SAE_: variability (standard deviation) of the shoulder angle error
YA: young adult group.
